# Ethnic differences in infectious burden and the association with metabolic risk factors for cardiovascular disease: a cross-sectional analysis

**DOI:** 10.1186/s12889-018-5162-x

**Published:** 2018-02-22

**Authors:** Lara Hartog, Martijn S. van Rooijen, Joanne Ujčič-Voortman, Maria Prins, Irene G. M. van Valkengoed

**Affiliations:** 1 Department of Public Health, Academic Medical Centre, University of Amsterdam, Meibergdreef 15, 1105 AZ, J2-209 Amsterdam, The Netherlands; 2 Department of Infectious Diseases, Public Health Service of Amsterdam, Amsterdam, The Netherlands; 3 Department of Documentation, Epidemiology and Health Promotion, Public Health Service of Amsterdam, Amsterdam, The Netherlands; 4 Division of Infectious Diseases, Department of Internal Medicine, Academic Medical Center (AMC), University of Amsterdam, Amsterdam, The Netherlands

**Keywords:** Infectious burden, Ethnicity, Diabetes, Hypertension, Hypercholesterolemia, Infections

## Abstract

**Background:**

The burden of metabolic risk factors for cardiovascular disease (CVD), such as type 2 diabetes, elevated cholesterol and hypertension, is unequally distributed across ethnic groups. Recent findings suggest an association of infectious burden (IB) and metabolic risk factors, but data from ethnic groups are scarce. Therefore, we investigated ethnic differences in IB and its association with metabolic risk factors.

**Methods:**

We included 440 Dutch, 320 Turkish and 272 Moroccan participants, 18–70 years, of the 2004 general health survey in Amsterdam, the Netherlands. IB was defined by seropositivity to the sum of 6 infections: Herpes Simplex Virus 1 and 2; Hepatitis A, B and C; and *Helicobacter pylori*. Associations between IB categories 4–6 (high), 3 (intermediate) and 0–2 (low) infections and metabolic risk factors were assessed by logistic regression. Finally, we determined the contribution of IB to the association between ethnicity and the metabolic risk factors by comparing adjusted logistic regression models with and without IB categories.

**Results:**

A high IB was more frequently observed among the Turkish and Moroccans than among the Dutch. After adjustment for age, sex, ethnicity, educational level, physical activity and body mass index, high IB was associated with type 2 diabetes (odds ratio high vs low IB (OR) =2.14, 95%-confidence interval (CI) 1.05–4.36). The association was weaker and not statistically significant, for elevated cholesterol (OR = 1.39, 95%-CI 0.82–2.34) and hypertension (OR = 1.49, 95%-CI 0.88–2.51). IB attenuated ethnic differences particularly for type 2 diabetes.

**Conclusions:**

Our study showed that Turkish and Moroccan adults in Amsterdam have a higher IB than Dutch adults, which was associated with the differences in type 2 diabetes. Due to the cross-sectional nature of the study, we cannot draw a conclusions with regards to the time-sequence of cause and effect. Nevertheless, the findings ask for further research into the nature of association of IB with metabolic risk factors in a longitudinal setting.

## Background

Cardiovascular disease (CVD) is the leading cause of death worldwide [1] and mortality rates are expected to increase [2]. The burden of CVD and its metabolic risk factors, such as type 2 diabetes mellitus (henceforth diabetes), elevated cholesterol levels and hypertension [3–5], is unequally distributed across populations. A higher prevalence is often found among ethnic minority populations in high income countries compared to the host population [6–10]. In the Netherlands, for instance, Turkish and Moroccan migrants have a higher prevalence of cardiovascular morbidity, diabetes and total/high-density lipoprotein (HDL)-cholesterol levels, but a lower prevalence of hypertension than the Dutch population [3–5]. These ethnic differences cannot be fully explained by conventional risk factors for CVD, such as socioeconomic position or obesity [3–5]. Research into alternative factors that might explain these disparities is, therefore, needed.

Numerous studies have shown the importance of infections by single infectious agents in the development of CVD [11–13]. However, recent studies suggest that the cumulative infectious burden (IB), may have an even bigger contribution to the development of CVD than individual infections [13, 14]. The mechanism is yet unclear, but one hypothesis is that multiple infections amplify each other’s effects on cardiovascular tissues [13]. Another proposed mechanism is that there is continuous stimulation of vascular inflammation caused by chronic infections or by a history of infections [15]. Indeed, studies have shown an association between a high IB and the prevalence or incidence of coronary artery disease [16], stroke [17] and atherosclerosis [13]. Some studies also showed an association between IB and the prevalence of metabolic risk factors of CVD, such as insulin resistance [18], diabetes [19], low HDL-cholesterol levels [20, 21], hypertension [22] and higher systolic- and diastolic blood pressure [19].

The IB may also play a role in the ethnic differences in metabolic risk factors for CVD as infection incidence and prevalence are known to differ across ethnic groups and geographic regions. For instance, two studies in the United States of America found that African Americans and Hispanics had a higher seroprevalence of infections such as hepatitis A virus (HAV) and *Helicobacter pylori* (*H.pylori)* compared to non-Hispanic whites [23, 24]. Additionally, in the United Kingdom, South Asians were found to have higher levels of serum gamma globulin, a non-specific measure of immune activation, compared to Europeans [25]. Information on burden of infection in other ethnic minority groups, such as Turkish and Moroccans, is lacking, though evidence shows that the prevalence of several individual infections is greater among these ethnic minority groups, compared to ethnic majority groups [26, 27]. Evidence on the possible contribution of IB to the ethnic differences in metabolic risk factors is also scant.

Therefore, we explored ethnic differences in IB and the association with metabolic risk factors of CVD in a study among 18 to 70-year old people of Dutch, Turkish and Moroccan origin in Amsterdam, the Netherlands. Specifically, we described ethnic differences in IB and analyzed the association of IB with prevalent diabetes, elevated cholesterol and hypertension. In addition, we assessed whether IB may contribute to the ethnic differences in prevalent diabetes, elevated cholesterol and hypertension.

## Methods

### Study population

The study population comprises participants of Dutch, Turkish and Moroccan descent from the population-based Amsterdam Health Monitor, a cross-sectional study conducted in 2004 by the Public health Service of Amsterdam in collaboration with the National Institute for Public Health and the Environment. Although inhabitants of other ethnic origin also participated, the current study was restricted to those of Turkish and Moroccan descent as they form the two largest non-Dutch ethnic groups in the Netherlands [28].

The methods of this study have been reported previously [3–5, 29]. In brief, a stratified random sample was drawn from the Amsterdam municipal registers in five city districts that were representative of the total population of Amsterdam. The sample was stratified by ethnicity and age (age groups: 18–34, 35–44, 45–54, 55–64 and > 65 years), Turkish and Moroccan respondents were oversampled, to ensure sufficient numbers in these groups [5]. The response to the invitation for the study was 44% (45.8%, 49.6% and 38.7% for the Dutch, Turkish and Moroccan population) [29]. In total, 1329 Dutch, Turkish and Moroccan people were included. Of this group, 79% of participants donated a blood sample. Thus, after exclusion of those with incomplete data on diabetes, elevated cholesterol, or hypertension (*n* = 10) and those for whom data on infections (*n* = 287) were not available due to insufficient amounts of blood, unclear results or no permission for blood storage, data on 1032 participants were available for analysis (440 Dutch, 320 Turkish and 272 Moroccans).

The study was approved by the Institutional Review Board of the Amsterdam Medical Centre of the University of Amsterdam [29]. Participants provided a written informed consent.

### Data collection

All participants took part in a structured interview on socio-demographic factors, lifestyle and health in their language of choice (Dutch, Turkish, Moroccan-Arabic or Berber). In addition, all participated in a physical examination conducted according to standardized protocols. Body height and weight were measured in light clothing without shoes. Height was measured to the nearest 0.5 cm with a wall mounted stadiometer and weight was measured to the nearest 0.5 kg with a calibrated analogue scale (SECA; SECA gmbh&co, Hamburg, Germany). For analysis, body weight was adjusted for the clothes worn by subtracting 1 kg from the measured weight. Blood pressure was measured by a validated oscillometric automated device (Omron HEM-711; OMRON Healthcare BV, Hoofddorp, the Netherlands). The reading was taken on the left arm with appropriate cuff sizes, after the participants had been seated for a minimum of 5 min. All measures were taken in duplicate and the mean of the two measurements was used for analysis [29].

At the time of the physical examination, non-fasting blood samples were collected for direct measurements and for storage. Glycated hemoglobin (HbA1c) levels, non-fasting glucose levels and total- and HDL-cholesterol levels were determined directly with standard laboratory techniques (Hitachi 911, Roche Diagnostics, Mannheim, Germany) at the Central Clinical Chemical Laboratory of the Erasmus Medical Centre in Rotterdam, the Netherlands [3, 4].

From the infections measured in the total population of the Amsterdam Health Monitor, we considered herpes simplex virus 1 (HSV1), herpes simplex virus 2 (HSV2), HAV, hepatitis B virus (HBV), hepatitis C virus (HCV) and *H. pylori* as these had been measured in the full population and had been included in previous definitions of IB based on their presumed associations with CVD [13–21]. Seropositivity to these infections was determined in stored samples by the Public Health Laboratory in Amsterdam as follows:

HSV1 and HSV2 were determined by antibody assays (HerpesSelect, Focus Technologies, USA) [26]. Normalized optical density (OD) readings were recorded, values above and including 1.1 OD were reported as positive, values below 0.9 were reported as negative [26]. In line with Zhu et al. [16], equivocal values were defined as HSV1/2-negative.

HAV, HBV and HCV were classified by the presence of antibodies to the antigens (anti-HAV, anti-HBc and anti-HCV, respectively) by means of microparticle enzyme immunoassays [HAVAB 2.0, Abbott/Axsym; CORE Abbott/Axsym; HCV version 3.0, Abbott/Axsym, respectively [27]]. Results that had been flagged as ambiguous due to mostly insufficient availability of material for analysis, were coded as missing values.

*H.pylori* was tested by means of *H.pylori* serology for the strains 26,695 and G27, specifically for antibodies against proteins: HP0073, HP0243, HP0010, HP1564, HP0547/1, HP0547/2, HP0887/1, HP0887/2, HP1098 and HP0659/1. Values above and including 200 MFI were defined as seropositive. This threshold was based on percentile distribution plots, since there was no external reference panel available. Seropositivity to at least 4 proteins was considered as *H.pylori* –positive, since this has shown good agreement (κ = 0.70) with serologic assay classification [30].

### Definitions

#### Ethnicity and covariates

Ethnicity was defined according to the definition of the Dutch Ministry of Internal Affairs by the self-reported country of birth of the participant or the participant’s father or mother [31, 32].

Highest level of education attained was used as a proxy for socio-economic position [4]. Low was defined as secondary school or lower.

Current cigarette smoking was defined according to self-reported smoking at time of the interview. Being physically active was defined according to the Dutch norm for healthy physical activity, as at least half an hour of moderate activity on at least 5 days a week [33]. Body mass index (BMI) was calculated by weight (kg) divided by squared height (m2).

#### Metabolic risk factor

Diabetes was defined by self-reported diabetes and/or by the use of anti-diabetic medication (oral hypoglycemic agents or insulin) and/or by a measured non-fasting glucose level of > 11.0 mmol/L and/or a measured HbA1c level of > 48 mmol/mol [3]. Elevated cholesterol was defined by self-reported high cholesterol and/or by the use of lipid lowering medication and/or by having a ratio greater than 5 for the total/HDL-cholesterol ratio, a threshold advised by Devroey et al. [34]. Hypertension was defined by self-reported hypertension and/or by the use of antihypertensive medication and/or by a measured systolic blood pressure of ≥140 mmHg and/or a diastolic blood pressure of ≥90 mmHg [5, 35].

#### Infectious burden

We defined IB as the total number of infections. With the term infection we here refer to antibody seropositivity, a measure reflecting current or past infection. For analysis, IB was divided into categories based on the distribution in the population (low: 0–2 infections, intermediate: 3 infections, and high: 4–6 infections).

### Statistical analysis

We used means (standard errors (SE)), medians (IQR), or n (percentages) to describe the baseline characteristics of participants in the three ethnic groups. Differences between the ethnic groups were calculated by chi square tests or Kruskal Wallis tests. We then described the IB and infection prevalence, and determined the age and sex-adjusted differences in high IB (versus the combined intermediate and low IB categories as a reference) and in individual infections with logistic regression.

Subsequently, we analyzed the association of IB categories with diabetes, elevated cholesterol and hypertension by means of logistic regression analysis. We adjusted for age, sex and ethnicity. In an additional analysis, we also adjusted for education level, physical activity and BMI. Moreover, we repeated the analysis after exclusion of people with a self-reported diagnosis. We reported all associations for the total population, as analysis of effect modification did not show evidence of a difference in association by ethnicity (*p* > 0.05 for the likelihood ratio test). We repeated these analyses for the individual infections.

Finally, to assess the possible contribution of IB to the ethnic differences in metabolic risk factors, we determined the association of ethnicity and the metabolic risk factors before and after adjustment for IB. For this analysis, we used logistic regression, with ethnicity as the indicator variable. We considered a change of more than 10 % in the estimate for the OR, regardless of statistical significance of the estimate, as potentially relevant. Analyses were adjusted for age, sex, education level, current cigarette smoking, physical activity and BMI. These were selected based on their known association with ethnicity and metabolic risk factors, e.g. from previous analyses within the study population [3–5].

All statistical tests were two-tailed and *P*-values of less than 0.05 were considered statistically significant. All analyses were performed using SPSS 20.0 for windows (SPSS Inc., Chicago, IL, USA).

## Results

Between 41% (Dutch) and 58% (Moroccan) of participants were men (Table 1). Dutch participants were older and higher educated than participants in the other ethnic groups. A minority of Turkish and Moroccan participants were born in the Netherlands. Current cigarette smoking was most common in the Dutch group, whereas alcohol abstinence was most common in the Turkish and Moroccan groups. Physical activity levels were lowest and BMI highest among the Dutch. The prevalence of diabetes was highest among Moroccans, followed by the Turkish and the Dutch. A similar pattern was observed for the underlying glucose measures. The prevalence of elevated cholesterol was also higher among Turkish and Moroccan participants compared to the Dutch participants, while total cholesterol and HDL-cholesterol levels were highest among the Dutch. The prevalence of hypertension and the measured blood pressure levels were higher among the Dutch than the Turkish and the Moroccans.

**Table 1:** Characteristics of the study population

	Dutch (*n* = 440)	Turkish (*n* = 320)	Moroccan (*n* = 272)	*P* value
Male	181 (41.1)	151 (47.2)	158 (58.1)	< 0.001
Mean age in years	51.5 ± 14.8	45.2 ± 13.2	49.7 ± 13.9	< 0.001
Low education level ^a^	77 (17.7)	193 (62.3)	177 (66.8)	< 0.001
Born in the Netherlands	428 (97.3)	13 (4.8)	13 (4.1)	< 0.001
Current cigarette smoking	141 (32.2)	105 (36.1)	40 (15.2)	< 0.001
Alcohol abstinence	40 (10.2)	211 (81.8)	198 (92.5)	< 0.001
Physically active > = 30 min on 5 days	306 (69.5)	138(43.1)	145 (53.3)	< 0.001
Mean body mass index in kg/m^2^	25.8 ± 4.5	28.9 ± 5.3	27.8 ± 5.3	< 0.001
Diabetes ^b^	35 (8.0)	62 (19.4)	71 (26.3)	< 0.001
-Median non-fasting blood glucose in mmol/L	4.9 (4.6–5.5)	5.1 (4.6–6.0)	5.5 (4.9–6.9)	< 0.001
-Median glycated hemoglobin in %	5.5 (5.3–5.8)	5.6 (5.3–5.9)	5.7 (5.4–9.1)	< 0.001
Elevated Cholesterol ^c^	106 (24.1)	122 (38.4)	81 (30.1)	< 0.001
-Median high-density lipoprotein cholesterol in mmol/L	1.5 (1.3–1.8)	1.2 (1.0–1.4)	1.2 (1.0–1.4)	< 0.001
-Mean total cholesterol in mmol/L	5.6 ± 1.1	5.2 ± 1.0	5.1 ± 1.0	< 0.001
Hypertension ^d^	223 (50.8)	130 (41.0)	108 (39.7)	0.001
-Mean systolic blood pressure in mmHg	137.8 ± 23.5	129.7 ± 22.1	133.2 ± 21.4	< 0.001
-Mean diastolic blood pressure in mmHg	82.8 ± 11.6	81.6 ± 11.2	79.7 ± 10.4	0.001

Data are given as n (%), mean ± sd or median (interquartile range); With the exception of low education level (*n* = 21), current cigarette smoking (*n* = 39), body mass index (*n* = 12) and alcohol abstinence (*n* = 166), eight or fewer participants had missing values for the specified variables; *P*-values for the differences between ethnic groups; ^a^ Low education level was measured as up to and including secondary school; ^b^ Diabetes was measured by self-report and/or glucose > 11.0 mmol/L and/or HbA1c > 6.5%; ^c^ Elevated cholesterol was measured by self-report and/or total/HDL-cholesterol ≥5 *mmol/L;* ^d^ Hypertension was measured by self-report and/or DBP ≥90 mmHg or SBP ≥140 mmHg

In total, 8.6% of the Dutch, 37.9% of the Moroccan and 41.1% of the Turkish participants fell into the high IB category (Table 2). The crude prevalence of HSV1, HAV, HBV and *H. pylori* showed a similar pattern of differences between the groups as the prevalence of high IB (Table 2). In contrast, HSV2 occurred less frequently among the Turkish and Moroccans than among the Dutch. After adjustment for age and sex, the differences between groups remained (e.g. odds ratio (OR) for high IB in Turkish versus Dutch: 10.8, 95%-confidence interval (CI) 7.0–16.5).

**Table 2:** Ethnic differences in the infectious burden and the prevalence of infections

	Crude prevalence			Age and sex adjusted OR (95%-CI)	
	Dutch (n = 440)	Moroccan (n = 272)	Turkish (n = 320)	Moroccan versus Dutch	Turkish versus Dutch
IB					
-High	38 (8.6)	103 (37.9)	138 (41.1)^a^	7.0 (4.6–10.8)^b^	10.8 (7.0–16.5)^b^
-Intermediate	110 (25.0)	155 (57.0)	165 (51.6)		
-Low	292 (66.4)	14 (5.1)	17 (5.3)		
HSV1 positive	281 (63.9)	269 (98.9)	308 (96.3)^a^	60.5 (18.9–193.3)	19.0 (10.2–35.5)
HSV2 positive	100 (22.7)	43 (15.8)	35 (10.9)^a^	0.68 (0.45–1.0)	0.45 (0.30–0.69)
HAV positive	217 (49.3)	261 (96.0)	310 (96.9)^a^	35.6 (18.4–69.2)	22.2 (27.6–110.5)
HBV positive	25 (5.7)	77 (28.3)	120 (37.5)^a^	6.8 (4.1–11.2)	13.6 (8.3–22.2)
HCV positive	1 (0.2)	1 (0.4)	0 (0)	–	–
*H.pylori* positive	252 (57.3)	267 (98.2)	313 (97.8)^a^	46.5 (18.6–116.0)	44.6 (20.2–98.2)

Data are given as n (%) or as median (minimum-maximum), *IB* infectious burden measured by the number of infections (HSV1, HSV2, HAV, HBV, HCV and *H.pylori)* a participant was seropositive for. With the term infection we in this study refer to antibody seropositivity, a measure reflecting current or past infection. The subcategories are: low (0–2 infections), intermediate (3 infections) and high (4–6 infections)*; HSV1* herpes simplex virus 1, *HSV2* herpes simplex virus 2, *HAV* hepatitis virus A, *HBV* hepatitis virus B, *HCV* hepatitis virus C, *H.pylori Helicobacter pylori, OR* odds ratio, *CI* confidence interval, ^a^ *p-value univariate comparison < 0.001,* ^b^ Comparison made between high IB and other

High IB appeared to be associated with diabetes; the age, sex and ethnicity adjusted OR for diabetes for high compared to low IB was 2.30 (95%-CI 1.14–4.65; Table 3). This association remained after further adjustment (data shown in Appendix 1). Moreover, associations were similar when self-reported cases were excluded (Appendix 2). Slightly elevated odds, albeit non-significant, were also observed for high IB in association with elevated cholesterol and hypertension. The estimates for the individual infections showed that, in particular, HSV2 was significantly associated with the metabolic risk factors.

**Table 3:** Associations between infectious burden, individual infections and metabolic risk factors

Infections	Diabetes^a^	Elevated cholesterol^b^	Hypertension^c^
	OR (95%-CI)	OR (95%-CI)	OR (95%-CI)
IB			
- High	**2.30 (1.14–4.65)**	1.54 (0.93–2.56)	1.60 (0.96–2.67)
- Intermediate	1.52 (0.78–2.97)	1.52 (0.97–2.39)	1.40 (0.89–2.20)
- Low	Reference	Reference	Reference
HSV1	1.36 (0.65–2.86)	0.80 (0.51–1.25)	1.11 (0.72–1.71)
HSV2	**1.96 (1.24–3.11)**	**1.77 (1.21–2.57)** ^*^	1.37 (0.94–2.00)
HAV	1.17 (0.59–2.34)	1.21 (0.77–1.89)	0.81 (0.53–1.24)
HBV	1.16 (0.75–1.80)	0.87 (0.59–1.26)	1.25 (0.85–1.82)
HCV^d^	–	–	–
*H.pylori*	1.57 (0.74–3.34)	1.29 (0.81–2.04)	1.14 (0.75–1.75)

Bold = significantly different from the reference category within the specific model, ^*^ Overall P-value of < 0.05 for the adjusted model; All models were adjusted for age, sex and ethnicity, *IB* infectious burden measured by the number of infections (HSV1, HSV2, HAV, HBV, HCV and *H.pylori)* a participant was seropositive for. With the term infection we in this study refer to antibody seropositivity, a measure reflecting current or past infection. The subcategories are: low (0–2 infections), intermediate (3 infections) and high (4–6 infections)*, HSV1* herpes simplex virus 1, *HSV2* herpes simplex virus 2, *HAV* hepatitis virus A, *HBV* hepatitis virus B, *HCV* hepatitis virus C, *H.pylori Helicobacter pylori,* ^a^ Diabetes was measured by self-report and/or glucose > 11.0 mmol/L and/or HbA1c > 6.5%, ^b^ Elevated cholesterol was measured by self-report and/or total/HDL-cholesterol ≥5 mmol/L, ^c^ Hypertension was measured by self-report and/or DBP ≥ 90 mmHg or SBP ≥ 140 mmHg, ^d^ Logistic regression was not performed, due to low amount of HCV positive participants

Adjustment for the IB categories attenuated the ethnic differences in diabetes and, only for Turkish, in elevated cholesterol (Table 4). For instance, the OR for diabetes for the Moroccans compared to the Dutch decreased from 3.50 (95%-CI 1.97–6.23) in the adjusted model to 2.76 (95%-CI 1.43–5.33) after addition of IB. For hypertension, addition of IB seemed further lower the odds for Moroccans in comparison to the Dutch.

**Table 4:** Associations between ethnicity and metabolic risk factors, adjusted for infectious burden categories

	Adjusted model^a^	Adjusted model + IB^b^
	OR (95%-CI)	OR (95%-CI)
Diabetes^d, c^		
Dutch	Reference	Reference
Moroccan	**3.51 (1.97–6.25)**	**2.78 (1.44–5.38)**
Turkish	**2.41 (1.32–4.42)**	**2.14 (1.03–4.20)**
Elevated cholesterol^e, d^		
Dutch	Reference	Reference
Moroccan	0.98 (0.63–1.51)	0.74 (0.44–1.25)
Turkish	**1.64 (1.07–2.52)**	1.30 (0.78–2.16)
Hypertension^f, e^		
Dutch	Reference	Reference
Moroccan	**0.46 (0.30–0.71)**	**0.39 (0.23–0.66)**
Turkish	0.68 (0.44–1.05)	0.60 (0.36–1.00)

Bold = significantly different from the reference group within the specified model, ^a^ Adjusted for age, sex, education level, current cigarette smoking, physical activity and BMI, ^b^ Adjusted for age, sex, ethnicity, education level, current cigarette smoking, physical activity, BMI and IB. *IB* infectious burden measured by the number of infections (HSV1, HSV2, HAV, HBV, HCV and *H.pylori)* a participant was seropositive for, categorized into low (0–2 infections), intermediate (3 infections) and high (4–6 infections). With the term infection we in this study refer to antibody seropositivity, a measure reflecting current or past infection., ^c^ Diabetes was measured by self-report and/or glucose > 11.0 mmol/L and/or HbA1c > 6.5%, ^d^ Elevated cholesterol was measured by self-report and/or total/HDL-cholesterol ≥5 mmol/L, ^e^ Hypertension was measured by self-report and/or DBP ≥ 90 mmHg or SBP ≥ 140 mmHg

## Discussion

### Summary of key findings

We found a higher IB among the 18 to 70-year old participants of Turkish and Moroccan origin in our study than among those of Dutch origin. This increased IB was significantly associated with a higher odds of diabetes, but the association was not as clear for elevated cholesterol and hypertension. In line with this finding, IB attenuated the ethnic disparities in diabetes.

### Discussion of key findings

As expected, the Turkish and Moroccans had a higher IB than the Dutch. This was in line with previous reports signaling a higher prevalence of individual infections in these groups than the Dutch [26, 27]. The findings are also in line with the ethnic differences in IB reported in two studies from the US [23, 24]. In both studies, the IB was lower among the largely European origin population than among the other ethnic groups. As the prevalence of several infections was also found to be higher among other populations originating from low income countries living in the Netherlands, such as the Surinamese, Antilleans and Egyptians [26, 27, 36], a higher IB may also be expected in these groups.

The finding that ethnic minorities have a higher IB than the general Dutch population is likely related to background prevalence in the country of origin, in combination with increased early life exposure before migration, or exposure during travel to their region of origin post migration. Both Turkey and Morocco are countries with an increased background prevalence of, for instance, HAV and HBV compared to the Netherlands, and have been reported to have lesser hygienic circumstances [37]. Differences in IB may also be partly explained by increased susceptibility related to a lower socioeconomic position of ethnic minority groups or by differences in proportion vaccinated for HBV [23]. Poor circumstances may subject populations to social stress, which can down regulate the immunity, thus making these groups more susceptible to infections [38]. Additionally, the transmission dynamics may also be important. The historically high infection rates among ethnic minorities may partly remain due to the fact that minority populations live and work together, even as their socioeconomic positions change [23].

The consistently positive association of IB with prevalent diabetes and the non-significantly positive associations with elevated cholesterol and hypertension in our population support a possible association between IB and these metabolic risk factors. Previous studies have been inconsistent. For example, several studies reported positive associations with diabetes and its clinical markers [18, 19], elevated cholesterol [20, 21] and hypertension [19, 22] while others reported no association of IB with these factors [39, 40]. The differences between other studies and ours may be due to several reasons. The differences may simply reflect differences between the populations studied. Previous studies often did not included ethnic minority groups or did not adjust for ethnic differences in the analyses, despite known ethnic differences in infections and in metabolic risk factors [3–5]. Another possible explanation might be the lack of a consistent IB definition. Different studies have, for various reasons, used different combinations of chronic or past infections to define the IB. In our study, for instance, we were unable to include several infections (e.g. *C. pneumoniae, cytomegalovirus* and Epstein-Barr virus were not measured) that have been associated with metabolic risk, and included by others in their definition of IB. On the other hand, we included data on HAV, HBV and HCV, which some but not all of these other studies included. The inconsistency across studies may also reflect the lack of insight into the exact mechanisms of risk and indicates the importance of further work to determine which infections or combinations of infections associate most strongly with metabolic risk.

Consistent with the lack of ethnic specific data on the association of IB with metabolic risk, there is also a lack of data on the possible contribution of IB to ethnic disparities in diabetes, elevated cholesterol and hypertension. Our findings suggest that IB may contribute to differences in, particularly, diabetes between the ethnic groups. If confirmed in larger studies among multiethnic populations, the potential implication is that reducing the burden of infections or timely initiation of treatment directed at diminishing or reversing its effects might contribute to reducing disparities between ethnic groups. The mechanisms by which IB works (e.g. epigenetic effects or inflammatory processes) should then be studied, as well as specific patterns of infections, currency and chronicity of infections and the association of these with incidence of metabolic risk factors. Unfortunately, the power of our study and availability of specific data limited a preliminary exploration of such specific patterns.

### Limitations

First and foremost, the cross sectional design of our study poses a major limitation. The proposed implications should be read with caution, as causal inferences cannot be made. For example, previous work shows that diabetes might also cause impaired host defenses and thus predispose to infections [41, 42]. Although the analysis that excluded self-reported cases, presumably those with a longer diabetes duration and potentially lower host defense, showed comparable results (Appendix 2) to the analysis in the full population, we cannot draw firm conclusions with regards to the time-sequences of cause and effect.

There are some further limitations that merit discussion. First, the overall response rate was relatively low (44%). However, we find it unlikely that selective response would change the interpretation of our findings as there were no significant differences between the respondents and the non-respondents in a previous analysis [43].

Second, we did not distinguish between past and chronic infection, and the estimated IB may have been flawed by the definition of our measures. As indicated previously, IB was based on seropositivity to antibodies (mostly IgG). Several potentially relevant chronic infections were not measured. Additionally, IgG antibodies only indicate prior infection (or vaccination), and not current infection, chronicity or duration of infection [24]. Additionally, we did not measure mode of transmission. Nevertheless, prior studies have also based their IB definitions on IgG antibodies, and there is no conclusive evidence that consideration of other markers of infection (e.g. endovascular or circulation DNA, antigens of pathogens) give more reliable results [42].

Third, as in many epidemiological studies, we partly defined our outcomes based on self-reported diagnoses, in line with previous analyses within the study population [3–5]. This could be problematic if differences in reporting occur. However, our restricted analyses (Appendix 2) suggest that this did not affect our results. In addition, our definition was based on a single measurement of e.g. fasting glucose and blood pressure. Although this applies similarly to all subgroups, more elaborate measurements, e.g. an oral glucose tolerance test for the identification of diabetes [44], and confirmation of diagnosis by a repeated measurement [45, 46], may have given more precise estimates of the prevalence across groups.

Finally, the results may have been affected by residual confounding. We were unable to adjust for potentially important factors, such as dietary intake, hygiene and (early life) living conditions in our analyses. In addition, numbers were too small to distinguish between generations. Moreover, we were not able to investigate whether treatment for infections (e.g. HCV [47]), the body’s immune response or the presence of the germ contributed to the associations found, which may have caused us to wrongly ascribe the results to the effects of the infections.

## Conclusions

In conclusion, our study showed that Turkish and Moroccan adults in Amsterdam have a higher IB than Dutch adults and that a higher IB was associated with differences in metabolic risk factors, in particular diabetes. Due to the cross-sectional nature of the study, we cannot draw any conclusions with regards to the time-sequences of cause and effect. Nevertheless, the findings ask for further research into the nature of association of IB with metabolic risk factors in a longitudinal setting, including investigations of dose-response relationships and an investigation of possible underlying mechanisms leading to the incidence of metabolic risk factors for CVD. If the association and time-sequence are confirmed, a reduction in IB or treatment directed at effects of IB may possibly be investigated as a target for reducing ethnic disparities in metabolic risk factors.

## Appendix 1

**Table 5:** Fully adjusted associations between infectious burden, individual infections and metabolic risk factors

Infections	Diabetes^a^	Elevated cholesterol^b^	Hypertension^c^
	OR (95%-CI)	OR (95%-CI)	OR (95%-CI)
IB			
- High	**2.14 (1.05–4.36)**	1.39 (0.82–2.34)	1.49 (0.88–2.51)
- Intermediate	1.41 (0.71–2.78)	1.39 (0.87–2.23)	1.28 (0.80–2.04)
- Low	Reference	Reference	Reference
HSV1	1.30 (0.61–2.75)	0.72 (0.45–1.15)	1.05 (0.68–1.63)
HSV2	**1.88 (1.19–2.96)**	**1.85 (1.25–2.72)**	1.39 (0.95–2.05)
HAV	1.10 (0.54–2.23)	1.12 (0.70–1.79)	0.81 (0.53–1.25)
HBV	1.19 (0.76–1.85)	0.83 (0.56–1.22)	1.30 (0.88–1.91)
HCV^d^	–	–	–
*H.pylori*	1.52 (0.71–3.27)	1.20 (0.74–1.92)	1.05 (0.68–1.63)

Bold = significantly different from the reference category within the specific model, Fully adjusted = adjusted for age, sex, ethnicity, education level, physical activity and BMI; *IB* infectious burden measured by the number of infections (HSV1, HSV2, HAV, HBV, HCV and *H.pylori)* a participant was seropositive for. With the term infection we in this study refer to antibody seropositivity, a measure reflecting current or past infection. The subcategories are: low (0–2 infections), intermediate (3 infections) and high (4–6 infections)*, HSV1* herpes simplex virus 1, *HSV2* herpes simplex virus 2, *HAV* hepatitis virus A, *HBV* hepatitis virus B, *HCV* hepatitis virus C, *H.pylori Helicobacter pylori,* ^a^ Diabetes was measured by self-report and/or glucose > 11.0 mmol/L and/or HbA1c > 6.5%, *b* Elevated cholesterol was measured by self-report and/or total/HDL-cholesterol ≥5 mmol/L, *c* Hypertension was measured by self-report and/or DBP ≥ 90 mmHg or SBP ≥ 140 mmHg; d: Logistic regression was not performed, due to low amount of HCV positive participants

## Appendix 2

**Table 6:** Associations between infectious burden and metabolic risk factors, after exclusion of self-reported cases

	Diabetes^a^	Cholesterol^b^	Hypertension^c^
	OR (95%-CI)	OR (95%-CI)	OR (95%-CI)
IB^d^			
- High	2.54 (0.67–9.67)	1.27 (0.70–2.31)	1.47 (0.82–2.65)
- Intermediate	0.99 (0.27–3.66)	1.26 (0.73–2.17)	1.45 (0.85–2.47)
- Low	Reference	Reference	Reference

With the term infection we in this study refer to antibody seropositivity, a measure reflecting current or past infection*;* a:Diabetes was determined by glucose > 11.0 mmol/L and/or HbA1c > 6.5%, after exclusion of people with self-reported diabetes; b: Elevated cholesterol was measured by total/HDL-cholesterol ≥5 mmol/L, after exclusion of people with self-reported dyslipidemia; d, c: Hypertension was measured by DBP ≥ 90 mmHg or SBP ≥ 140 mmHg, after exclusion of people with self-reported hypertension; d: IB = infectious burden measured by the number of infections (HSV1, HSV2, HAV, HBV, HCV and *H.pylori)* a participant was seropositive for. With the term infection we in this study refer to antibody seropositivity, a measure reflecting current or past infection. The subcategories are: low (0–2 infections), intermediate (3 infections) and high (4–6 infections)

## Abbreviations

BMI: Body mass index
CI: Confidence interval
CVD: Cardiovascular disease
H.pylori: *Helicobacter pylori*
HAV: Hepatitis A virus
HBV: Hepatitis B virus
HCV: Hepatitis C virus
HDL: High-density lipoprotein
HSV1: Herpes simplex virus type 1
HSV2: Herpes simplex virus type 2
IB: Infectious burden
OR: Odds ratio
